# Adherence to Pharmacotherapy in Patients With Parkinson's Disease Taking Three and More Daily Doses of Medication

**DOI:** 10.3389/fneur.2019.00799

**Published:** 2019-07-31

**Authors:** Igor Straka, Michal Minár, Matej Škorvánek, Milan Grofik, Katarína Danterová, Ján Benetin, Egon Kurča, Andrea Gažová, Veronika Boleková, Kathryn A. Wyman-Chick, Ján Kyselovič, Peter Valkovič

**Affiliations:** ^1^Second Department of Neurology, Comenius University in Bratislava Faculty of Medicine, University Hospital Bratislava, Bratislava, Slovakia; ^2^Department of Neurology, Pavol Jozef Safarik University in Kosice Faculty of Medicine, University Hospital of Louis Pasteur, Košice, Slovakia; ^3^Department of Neurology, Comenius University in Bratislava Jessenius Faculty of Medicine in Martin, University Hospital Martin, Martin, Slovakia; ^4^Department of Neurology, University Hospital Bratislava, Slovak Medical University, Bratislava, Slovakia; ^5^Comenius University in Bratislava Faculty of Medicine, Institute of Pharmacology and Clinical Pharmacology, Bratislava, Slovakia; ^6^Pan-European University Faculty of Psychology, Institute of Clinical Psychology, Bratislava, Slovakia; ^7^HealthPartners Neuroscience Center, Saint Paul, MN, United States; ^8^Fifth Department of Internal Medicine, Comenius University in Bratislava Faculty of Medicine, University Hospital Bratislava, Bratislava, Slovakia; ^9^Centre of Experimental Medicine, Institute of Normal and Pathological Physiology, Slovak Academy of Sciences, Bratislava, Slovakia

**Keywords:** adherence, antiparkinson drugs, non-motor symptoms, Parkinson's disease, quality of life

## Abstract

**Background:** Once-daily treatment formulation is associated with better adherence in comparison to more complex medication regimens. The study aimed to detect the extent of adherence to pharmacotherapy in Parkinson disease (PD) patients who take a minimum of three daily doses of drugs, and to identify factors associated with lower levels of adherence.

**Methods:** The cohort was selected from non-demented PD patients. The 8-Item Morisky Medication Adherence Scale (MMAS-8), 8-Item Parkinson's Disease Questionnaire (PDQ-8), Geriatric Depression Scale (GDS), Non-Motor Symptom Assessment Scale (NMSS), 9-Item Wearing-off Questionnaire (WOQ-9), MDS-UPDRS III (motor examination), and IV (motor complications) scales were used in this study.

**Results:** From a total of 124 subjects, 33.9% reported a high level of adherence, 29.8% reported a medium level of adherence, and 36.3% reported a low level of adherence to their pharmacotherapy. The level of non-adherence correlated with gender, longer disease duration, higher scores of PDQ-8, NMSS, WOQ-9, and MDS-UPDRS IV. Detailed analysis of NMSS demonstrated a correlation between the level of adherence and domains sleep/fatigue, mood/cognition, perceptual problems/hallucinations, attention/memory, and urinary symptoms. Independent risk factors for non-adherence were excessive daytime sleepiness, anhedonia, and forgetfulness.

**Conclusion:** Non-adherence to more complicated medication regimens is frequent in PD patients and is associated with gender, longer PD duration, poorer quality of life, frequency and severity of non-motor symptoms, and more severe motor and non-motor fluctuations. Non-adherence was predicted by non-motor symptoms including fatigue, mood disturbances, and subjective cognitive complaints.

## Introduction

Parkinson's disease (PD) is a complex neurodegenerative disorder with various motor and non-motor symptoms. The goal of dopamine replacement therapy (L-DOPA, dopamine agonists, monoamine oxidase B inhibitors and catechol-O-methyltransferase inhibitors) is to achieve good clinical outcomes and to delay or alleviate long-term complications, particularly dyskinesias, and fluctuations in motor and non-motor symptoms. Pharmacologic treatment of non-motor symptoms (NMS) includes antidepressants, anxiolytics, antidementive agents, hypnotics, analgesics, drugs for autonomic dysfunction, and others (1). Adherence to pharmacotherapy describes the extent to which patient's behavior agrees with medical instruction of their physician (2). Non-adherence to pharmacotherapy is one of the most important medicine-related problems among patients with chronic conditions and is associated with a reduced quality of life (QoL), increased social burden, and higher health care costs. Unfortunately, physicians often underestimate the extent of non-adherence of their patients (3, 4). In PD patients, suboptimal adherence varies between 10 and 67% (5). A once-daily treatment regimen is associated with higher adherence in comparison to more complex regimens (6). With disease progression, the majority of PD patients use three or more daily doses of PD medications. We did not find consistency in the existing body of research concerning adherence to pharmacotherapy specifically among PD patients and there is a lack of research examining patients who are prescribed multiple daily doses of PD medications. The aim of this study was to (1) detect the extent of adherence to pharmacotherapy in patients with PD who take three or more daily doses of dopaminergic drugs, and (2) to identify factors associated with non-adherence.

## Materials and Methods

### Participants

We included 124 consecutive subjects (72 men and 52 women) with clinically established or clinically probable idiopathic PD diagnosed according to the MDS Clinical Diagnostic Criteria for PD (7). Our study was cross-sectional. Subjects were recruited from movement disorders outpatient departments of University Hospitals in Bratislava, Martin, and Košice (Slovakia). Study procedures were performed according to the declaration of Helsinki, ethical aspects were approved by local ethical committee for all centers, and all subjects signed written informed consent prior to inclusion into the study. We included patients without cognitive impairment (>26/30 points on the Mini Mental State Examination), on standard dopaminergic therapy with L-DOPA and/or dopamine agonists in minimum three daily doses (at stable doses and regimen for minimum of the last 4 weeks). Drugs for NMS were considered as PD therapy in the study context. Patients with four or more treated comorbidities (at stable doses and regimen for minimum of the last 4 weeks) were excluded from this study. Patients undergoing surgical or pump therapy for PD were also excluded.

### Methods

All patients were examined using validated Slovak translations of the following screening and diagnostic instruments:

- Medication adherence was measured by the 8-Item Morisky Medication Adherence Scale (MMAS-8) (8, 9), cut-offs for levels of MMAS-8 were stratify 0 points for high level of adherence, 1–2 points for medium level of adherence, and 3–8 points for low level of adherence (8);
- QoL was measured by the 8-Item Parkinson's Disease Questionnaire (PDQ-8) (10, 11);
- Depression was measured by the Geriatric Depression Scale (GDS) (12–14);
- Frequency and severity of NMS was measured by the Non-Motor Symptom Assessment Scale for PD (NMSS) (11, 15);
- Motor impairment was measured by the MDS—Unified Parkinson's Disease Rating Scale (MDS-UPDRS)—part III: motor examination (MDS-UPDRS III),
- Motor complications were measured by the MDS-UPDRS part IV: motor complications (MDS-UPDRS IV) (16, 17),
- Wearing-off phenomenon (defined as motor and non-motor symptom fluctuations) was measured by the 9-Item Wearing-off Questionnaire (WOQ-9) (18, 19).

In all scales and questionnaires, higher scores were associated with more severe symptoms.

The MMAS-8, PDQ-8, and WOQ-9 were completed by patients or caregivers. The GDS, NMSS, and MDS-UPDRS III, IV were administered by trained investigators. Baseline characteristics (age, disease duration, previous medication history, comorbidities), and Hoehn & Yahr score (H&Y) were recorded by patients and checked by investigators.

### Statistical Analysis

Statistical analysis of the data was performed using IBM SPSS Statistics 24. Demographic and clinical parameters were analyzed using descriptive statistics. Data in our cohort according to Kolmogorov-Smirnov test were non-parametric. The strength of the relationship between the parameters was measured by Spearman's rank correlation coefficient (r_s_) and correlation ratio eta (η). Stepwise multiple regressions were performed to study the relationship between the MMAS-8 and observed parameters. The level of statistical significance was set at *p* ≤ 0.05.

## Results

Based on MMAS-8 scores, 42 patients (33.9%; MMAS-8 = 0 points) reported a high level of adherence, 37 patients (29.8%; MMAS-8 = 1–2 points) reported a medium level of adherence, and 45 patients (36.3%; MMAS-8 ≥ 3 points) reported a low level of adherence.

Demographic and clinical data of our cohort are presented in Table 1. The scores of non-adherence to PD medication (according to the MMAS-8) positively correlated with disease duration, QoL, depression, frequency and severity of NMS, motor and non-motor complications. We observed lower adherence in male subjects. We did not find any effect of educational attainment on the level of adherence (Table 2). Also, we did not find significant differences in LEDD and MDS-UPDRS III between the groups of high, medium, and low adherent patients in our cohort.

**Table 1:** Demographic and clinical data.

	**All participants**	**High level of adherence**	**Medium level of adherence**	**Low level of adherence**
	**(*n* = 124)**	**(*n* = 42)**	**(*n* = 37)**	**(*n* = 45)**
Men	72 (58.1)	21 (50)	18 (48.6)	33 (73.3)
Women	52 (41.9)	21 (50)	19 (51.4)	12 (26.7)
Age	68 (11.8)	65.5 (11.3)	72 (9)	68 (14)
Secondary education	73 (58.9)	26 (61.9)	20 (54.1)	27 (60)
University education	51 (41.1)	16 (38.1)	17 (45.9)	18 (40)
Duration of PD	7 (5)	5 (5)	6 (3)	8 (6)
H&Y	2.5 (1)	2 (1)	2.5 (1)	2.5 (1)
LEDD	1089 (775.4)	1053.5 (827.9)	1007 (886.5)	1175 (671.3)
Number of PD drugs/day	9 (6)	8 (7)	9 (5)	9 (5.5)
Number of PD doses/day	5 (2)	5 (2)	5 (2)	5 (1)
MMAS-8	1.5 (3)	0 (0)	1 (1)	4 (2)
PDQ-8	8 (8)	7 (6)	8 (9)	11 (9)
GDS	8 (8)	6.5 (8.3)	7 (7.5)	10 (9)
NMSS	59 (48.8)	45.5 (44)	55 (37.5)	77 (54)
MDS-UPDRS III	30.5 (10)	29.5 (13.3)	30 (10)	32 (8)
MDS-UPDRS IV	5 (9)	3 (8)	6 (9)	7 (8)
WOQ-9	3 (3)	3 (3)	3 (3.5)	4 (2)

*Numerical variables were expressed as median (interquartile range), categorical variables (gender, education) were presented as n (%). GDS, Geriatric Depression Scale; H&Y, Hoehn and Yahr stage; LEDD, levodopa equivalent daily dose (mg); MMAS-8, 8-Item Morisky Medication Adherence Scale; MDS-UPDRS III, MDS-Unified Parkinson's Disease Rating Scale—part III: Motor examination; MDS-UPDRS IV, MDS-Unified Parkinson's Disease Rating Scale—part IV: Motor complications; NMSS, Non-Motor Symptom Assessment Scale; PD, Parkinson's disease; PDQ-8, 8-Item Parkinson's Disease Questionnaire; WOQ-9, 9-Item Wearing-off Questionnaire*.

**Table 2:** Spearman's rank correlation coefficient (r_s_) of non-adherence to PD medication (MMAS-8) and other rated variables.

	**r_**s**_**	***p***
Gender	0.189 (η)	0.045*
Age	0.009	0.923
Education	0.006 (η)	0.948
Duration of PD	0.241	0.007**
H&Y	0.158	0.080
LEDD	0.071	0.436
Number of PD drugs/day	0.013	0.889
Number of PD doses/day	0.066	0.466
PDQ-8	0.234	0.009**
GDS	0.279	0.002**
NMSS	0.343	<0.001**
MDS-UPDRS III	0.151	0.094
MDS-UPDRS IV	0.234	0.009**
WOQ-9	0.277	0.002**

*Gender and education were presented as correlation ratio eta (η). GDS, Geriatric Depression Scale; H&Y, Hoehn and Yahr stage; LEDD, levodopa equivalent daily dose; MMAS-8, 8-Item Morisky Medication Adherence Scale; MDS-UPDRS III, MDS-Unified Parkinson's Disease Rating Scale – part III: Motor examination; MDS-UPDRS IV, MDS-Unified Parkinson's Disease Rating Scale – part IV: Motor complications; NMSS, Non-Motor Symptom Assessment Scale; PD, Parkinson's disease; PDQ-8, 8-Item Parkinson's Disease Questionnaire; WOQ-9, 9-Item Wearing-off Questionnaire*.

*Bold is used to highlight statistical significance*,

* *p ≤ 0.05*,

** *p < 0.01*.

Only the frequency and severity of NMS predicted lower levels of adherence to pharmacotherapy (Table 3). From detailed analysis of NMS (based on NMSS scale), we found out significant correlations between the scores of non-adherences and domains sleep/fatigue, mood/cognition, perceptual problems/hallucinations, attention/memory, and urinary symptoms (Table 4). Analysis of isolated questions in NMSS scale of above-mentioned domains reported significant correlations with items 3–4, 10–14, 16–18, and 24 of the MMAS-8 (Table 5). In the stepwise multiple regressions, we confirmed that only: excessive daytime sleepiness, anhedonia, and forgetfulness (items 3, 12, and 18) predicted worse adherence to PD medication (Table 6).

**Table 3:** Linear regression analysis of non-adherence to PD medication (MMAS-8) and factors associated with poorer adherence.

	**Unstandardized coefficients**		**Standardized coefficients**	**t**	***p***	**95% CI**
	**B**	**Std. error**	**Beta**			
Duration of PD	0.043	0.034	0.112	1.264	0.209	−0.024–0.109
PDQ-8	0.029	0.034	0.087	0.843	0.401	−0.039–0.097
GDS	0.020	0.032	0.064	0.612	0.542	−0.044–0.084
NMSS	0.014	0.005	0.290	2.883	0.005**	0.005–0.024
MDS-UPDRS IV	−0.024	0.042	−0.066	−0.575	0.566	−0.107–0.059
WOQ-9	0.117	0.085	0.144	1.378	0.171	−0.051–0.284
R = 0.448, R^2^ = 0.201, ${\text{R}}_{adj}^{2}$ = 0.160, Standard error of the estimate = 1.650						
Dependent variable: MMAS-8						

*GDS, Geriatric Depression Scale; MMAS-8, 8-Item Morisky Medication Adherence Scale; MDS-UPDRS IV, MDS-Unified Parkinson's Disease Rating Scale—part IV: Motor complications; NMSS, Non-Motor Symptom Assessment Scale; No, number; PD, Parkinson's disease; PDQ-8, 8-Item Parkinson's Disease Questionnaire; WOQ-9, 9-Item Wearing-off Questionnaire*.

Bold is used to highlight statistical significance, *p ≤ 0.05,

** *p < 0.01*.

**Table 4:** Spearman's rank correlation coefficient (r_s_) of non-adherence to PD medication (MMAS-8) and domains of NMSS scale.

	**r_**s**_**	***p***
1. Cardiovascular including falls	0.135	0.134
2. Sleep/fatigue	0.178	0.048*
3. Mood/cognition	0.262	0.003**
4. Perceptual problems/hallucinations	0.195	0.030*
5. Attention/memory	0.480	<0.001**
6. Gastrointestinal tract	0.133	0.140
7. Urinary	0.218	0.015*
8. Sexual function	0.129	0.164
9. Miscellaneous	0.166	0.065

*MMAS-8, 8-Item Morisky Medication Adherence Scale; NMSS, Non-Motor Symptom Assessment Scale*.

*Bold is used to highlight statistical significance*,

* *p ≤ 0.05*,

** *p < 0.01*.

**Table 5:** Spearman's rank correlation coefficient (r_s_) of non-adherence to PD medication (MMAS-8) and questions of relevant domains of NMSS scale.

	**r_s_**	***p***
**Domain 2: Sleep/fatigue**		
3. Does the patient doze off or fall asleep unintentionally during daytime activities?	0.354	<0.001**
4. Does fatigue (tiredness) or lack of energy (not slowness) limit the patient's daytime activities?	0.348	<0.001**
5. Does the patient have difficulties falling or staying asleep?	0.005	0.952
6. Does the patient experience an urge to move the legs or restlessness in legs that improves with movement when he/she is sitting or lying down inactive?	0.013	0.889
**Domain 3: Mood/cognition**		
7. Has the patient lost interest in his/her surroundings?	0.031	0.736
8. Has the patient lost interest in doing things or lack motivation to start new activities?	0.073	0.418
9. Does the patient feel nervous, worried, or frightened for no apparent reason?	0.087	0.338
10. Does the patient seem sad or depressed or has he/she reported such feelings?	0.342	<0.001**
11. Does the patient have flat moods without the normal “highs” and “lows?”	0.234	0.009**
12. Does the patient have difficulty in experiencing pleasure from their usual activities or report that they lack pleasure?	0.285	0.001**
**Domain 4: Perceptual problems/hallucinations**		
13. Does the patient indicate that he/she sees things that are not there?	0.227	0.011*
14. Does the patient have beliefs that you know are not true?	0.181	0.044*
15. Does the patient experience double vision?	0.003	0.975
**Domain 5: Attention/memory**		
16. Does the patient have problems sustaining concentration during activities?	0.362	<0.001**
17. Does the patient forget things that he/she has been told a short time ago or events that happened in the last few days?	0.458	<0.001**
18. Does the patient forget to do things?	0.427	<0.001**
**Domain 7: Urinary**		
22. Does the patient have difficulty holding urine?	0.201	0.025*
23. Does the patient have to void within 2 h of last voiding?	0.100	0.271
24. Does the patient have to get up regularly at night to pass urine?	0.196	0.029*

*MMAS-8, 8-Item Morisky Medication Adherence Scale; NMSS, Non-Motor Symptom Assessment Scale*.

*Bold is used to highlight statistical significance*,

* *p ≤ 0.05*,

** *p < 0.01*.

**Table 6:** Stepwise multiple regression analysis model of predictors of non-adherence to PD medication (MMAS-8) and relevant questions of scale NMSS.

**Model**	**Question**	**Unstandardized coefficients**		**Standardized coefficients**	**t**	***p***	**95% CI**
		**B**	**Std. Error**	**Beta**			
1	17	0.342	0.056	0.487	6.166	<0.001**	0.233–0.452
2	17	0.217	0.071	0.308	3.063	0.003**	0.077–0.357
	18	0.231	0.084	0.278	2.762	0.007**	0.066–0.397
3	17	0.152	0.071	0.216	2.121	0.036*	0.010–0.293
	18	0.272	0.082	0.327	3.321	0.001**	0.110–0.434
	12	0.227	0.073	0.242	3.110	0.002**	0.083–0.372
4	17	0.122	0.072	0.174	1.705	0.091	-0.020–0.264
	18	0.237	0.082	0.285	2.880	0.005**	0.074–0.400
	12	0.228	0.072	0.243	3.172	0.002**	0.086–0.371
	3	0.129	0.058	0.179	2.214	0.029*	0.014–0.245
Model 1: R = 0.487, R^2^ = 0.238, ${\text{R}}_{adj}^{2}$ = 0.213, Standard error of the estimate = 1.578							
Model 2: R = 0.532, R^2^ = 0.283, ${\text{R}}_{adj}^{2}$ = 0.271, Standard error of the estimate = 1.537							
Model 3: R = 0.580, R^2^ = 0.336, ${\text{R}}_{adj}^{2}$ = 0.320, Standard error of the estimate = 1.485							
Model 4: R = 0.602, R^2^ = 0.363, ${\text{R}}_{adj}^{2}$ = 0.341, Standard error of the estimate = 1.461							
Dependent variable: MMAS-8							

*Questions: 3—Does the patient doze off or fall asleep unintentionally during daytime activities? 12—Does the patient have difficulty in experiencing pleasure from their usual activities or report that they lack pleasure? 17—Does the patient forget things that he/she has been told a short time ago or events that happened in the last few days? 18—Does the patient forget to do things? MMAS-8, 8-Item Morisky Medication Adherence Scale; NMSS, Non-Motor Symptom Assessment Scale*.

*Bold is used to highlight statistical significance*,

* *p ≤ 0.05*,

** *p < 0.01*.

## Discussion

### General Aspects of Adherence to PD Medication

Results of our study indicate that non-adherence to pharmacotherapy in PD patients who take at least three daily doses of medication is prevalent. These findings are consistent with previous studies of PD patients (1, 6, 20–27) but none of the existing studies specifically examined patients with more complex therapeutic regimens. The level of adherence to medications correlated with PD disease duration, which is consistent with previous research (6, 22–24); however, in our sample, longer disease duration did not predict lower adherence. This may be due to the fact that non-adherence is widespread phenomenon at all stages of PD. In our study and in a previous study by Leopold et al. (22), lower adherence was observed in male subjects, while other studies have not demonstrated a relationship between the level of adherence and gender (1, 6, 23, 24, 28). Among PD patients who take at least three daily doses of PD medication, we did not find significant correlation between the level of adherence and levodopa equivalent daily dose, number of PD drugs, or number of doses per day. Previous research has focused on differences between medication adherence of once-daily formulations of dopamine agonists vs. multiple-daily formulations of medication. For example, a Multicentre European Study showed that a once-daily medication regimen had significantly higher overall and time adherence compared to more complex regimens (6). However, a study comparing extended-release and immediate-release dopamine agonist formulation did not demonstrate significant differences between the formulations (26).

### Level of Adherence to Pharmacotherapy in PD Is Linked to Non-motor Symptoms

We emphasize correlations between the level of adherence and NMS. Neuropsychiatric domains of the NMS had the strongest relationship to medication adherence. Specifically, sleep/fatigue, mood/cognition, perceptual problems/hallucinations, attention/memory, and urinary problems were significantly correlated with lower levels of adherence. In our sample, sleep attacks, anhedonia, and forgetfulness predicted lower adherence to PD medication. Fatigue is one of the most common and disabling symptoms and occurs in every stage of PD, which affects everyday activities, increases disability and reduces QoL (29, 30). Based on the results of our study, excessive daytime sleepiness was an important predictor of non-adherence in PD patients. Depression is a common NMS in PD. Patients with depression have worse adherence to medication (6, 31). We found that anhedonia was independent risk factor of lower adherence. Therefore, identifying and managing depressive symptoms may improve medication adherence (32). An association between non-adherence and cognitive impairment has been described in several studies (23, 25). In our sample, we found that the presence of subjective cognitive complaints significantly predicted non-adherence. The association between non-adherence and urinary symptoms is interesting. Patients with urinary symptoms have more severe motor symptoms (especially bradykinesia and axial symptoms), more frequent falls, and worse cognition than those without urinary symptoms (33, 34). These mechanisms are currently unexplained and future research is needed.

### Disease Progression Characteristics and Adherence to Pharmacotherapy in PD Patients

Therapeutic goals in the early stages of PD include control of motor symptoms and prevention of motor complications. In advanced stages of PD, they include management of motor complications and non-motor symptoms (4, 23). Antiparkinsonian drugs are usually prescribed multiple times per day. As the disease progresses, the medication regimen often includes complicated dosing schedules which are required to manage worsening motor symptoms. Non-adherence is also associated with worsening of motor symptoms and motor complications. Side effects or a poor response to PD medication are also associated with poorer adherence (4–6, 21, 23, 27). Worse baseline motor scores in early treated PD do not impair adherence and, on the contrary, they are associated with better adherence (28). In our cohort, motor scores did not correlate with level of adherence. This could be explained by the fact that our study included PD patients who were relatively advanced in the disease stage and from tertiary movement disorders departments. Non-adherence to PD medication is associated with more severe motor and non-motor complications (5, 23, 27). The MMAS-8 focuses on the miss of taking or forgetting to take the daily medication. However, our study design did not allow for definite identification as to the reason or result and to establish causality. Low levels of adherence may lead to changes in therapy because the physician (and patient) may attribute worsening of Parkinsonian symptoms (or subcompensation of PD) to lack of efficacy of PD medication and it may result to changes in drug schedules, doses, or drugs. This can generate adverse effects (including motor complications as well) and may deteriorate adherence to pharmacotherapy even further (6, 23, 27). However, it is very important to emphasize that in advanced stages of the disease, omission of a dose when dyskinesia is present or an extra dose of PD medication in the presence of a sudden wearing off of medication can improve the patient's current clinical condition.

### Quality of Life and Adherence to Pharmacotherapy in PD Patients

Consistent with previous research, non-adherent patients reported significantly poorer QoL (21, 23, 25, 35). Notably, the European Multicentre Study did not find poorer overall QoL in patients with suboptimal adherence and only the PDQ-39 mobility subscore was significantly associated with reduced QoL (6).

### Limitations

One limitation of our study was the usage of a subjective questionnaire for the detection of the level of medication adherence as opposed to objective medication monitoring methods. However, we utilized questionnaires that were specifically designed to assess medication adherence (36). Furthermore, the use of questionnaires mimics patient reports of medication adherence in clinical practice. Another limitation that may have impacted on our results was the selection of patients who take in a minimum of three daily doses of PD medication eliminating those with simpler therapeutic schemes.

## Conclusion

In summary, non-adherence to more complicated medication regimens is frequent among PD patients. In patients who take three and more daily doses of PD drugs, further increase in the number of daily doses was not connected to increased risk of impaired adherence. Moreover, age did not have effect on level of adherence to pharmacotherapy in non-demented PD patients. On the other hand, patients with longer duration of PD and males had significantly lower adherence. Non-adherence was associated with poorer QoL, non-motor symptoms and more severe motor and non-motor complications. We discovered that motor symptoms, especially excessive daytime sleepiness, anhedonia, and forgetfulness, were independent risk factors for lower levels of adherence. Awareness of suboptimal adherence is crucial in everyday clinical practice. The MMAS-8 self-report questionnaire is an easy tool which may allow clinicians to detect non-adherence to PD medication and provide education and intervention. Strategies, such as reminders, alarms set on mobile phones or digital wrist watches, may be helpful tools for improving medication adherence.

## Data Availability

The datasets generated for this study are available on request to the corresponding author.

## Ethics Statement

The studies involving human participants were reviewed and approved by Ethics Committee of Academic Derer's University Hospital Bratislava, University Hospital Bratislava. The patients/participants provided their written informed consent to participate in this study.

## Author Contributions

PV, JB, JK, AG, and IS: project conception and organization. IS, PV, MŠ, KD, and MG: clinical examinations. VB, MM, and IS: statistical analysis. IS, MM, and KW-C: manuscript writing. PV, MŠ, JB, EK, JK, AG, KD, MG, and VB: manuscript review and critique.

### Conflict of Interest Statement

The authors declare that the research was conducted in the absence of any commercial or financial relationships that could be construed as a potential conflict of interest. The funders had no role in study design, data collection, or preparation of the manuscript.
